# A multiresolution framework for the analysis of community structure in international trade networks

**DOI:** 10.1038/s41598-023-32686-2

**Published:** 2023-04-07

**Authors:** Wonguk Cho, Daekyung Lee, Beom Jun Kim

**Affiliations:** 1 Graduate School of Data Science, Seoul National University, Seoul, 08826 Republic of Korea; 2 Department of Physics, Sungkyunkwan University, Suwon, 16419 Republic of Korea; 3 Department of Energy Technology, Korea Institute of Energy Technology, Naju, 58322 Republic of Korea

**Keywords:** Complex networks, Statistical physics

## Abstract

International trade networks are complex systems that consist of overlapping multiple trade blocs of varying sizes. However, the resulting structures of community detection in trade networks often fail to accurately represent the complexity of international trade. To address this issue, we propose a multiresolution framework that integrates information from a range of resolutions to consider trade communities of different sizes and reveal the hierarchical structure of trade networks and their constituent blocks. In addition, we introduce a measure called multiresolution membership inconsistency for each country, which demonstrates the positive correlation between a country’s structural inconsistency in terms of network topology and its vulnerability to external intervention in terms of economic and security functioning. Our findings show that network science-based approaches can effectively capture the complex interdependencies between countries and provide new metrics for evaluating the characteristics and behaviors of countries in both economic and political contexts.

## Introduction

The international trade network is a complex system largely regulated by preferential trade agreements (PTAs), or trade blocs where preferential access to certain products is given between participating countries ^1^. In theory, any random pair of countries can sign a PTA as long as it is beneficial for their economies, but in reality PTAs do not spread uniformly. The findings of Manger et al. ^2^ suggest that structural arbitrage effects in trade networks lead to an endogenous spread of PTAs, with some countries making a multitude of deals while others remain relatively uninvolved.

In network theory, we define a group of nodes that are more densely connected with nodes within the group as a community ^3,4^. Community detection methods can be used to find a community structure in trade networks and identify which countries are more involved with each other. However, the conventional approach is fundamentally limited to the choice of a resolution parameter ^5–7^. That is, it can only identify a snapshot of trade communities from a certain range of sizes based on a given resolution. The dependence on the resolution parameter leads to a failure of reflecting the complex landscape of international trade, where multiple trade blocs with varying sizes coexist and overlap with one another.

On the other hand, applying community detection methods to trade networks is also susceptible to the inconsistency problem. That is, the resulting structures of community detection often show considerable variation, which is typically considered as evidence that the community detection methods are unreliable ^8–11^. In order to address this issue, recent studies have suggested alternative approaches that focus on, instead of obtaining the single best division among high-score divisions, analyzing how similar those divisions are ^12–14^. Indeed, Riolo and Newman ^12^ demonstrate that seemingly inconsistent divisions can be assembled to identify a set of underlying subgroups of nodes (building blocks) that consistently share the same community membership across different divisions.

Furthermore, an attempt to quantify the extent of inconsistency in different divisions was made by Lee et al. ^14^, suggesting a method for measuring the inconsistency of community structures in each level of resolution. By applying their framework to different networks, they discover that the community structures of a resolution with high consistency not only remain relatively persistent when changing the resolution but also have a better representation of the true division.

In this paper, we address the importance of incorporating information from inconsistent divisions across different resolutions and within each resolution. In real networks, particularly in international trade networks, each node belongs to multiple communities of different scales. Theses overlapping communities often lead to the construction of hierarchical structures, where nodes that belong to the same community at a small scale also share the same community membership at a larger scale. Therefore, in order to fully understand the structural properties of nodes, it is necessary to examine the consistency of each pair of nodes across a range of scales.

To address this issue, we propose a multiresolution framework that incorporates information from inconsistent divisions at various resolutions. Our multiresolution framework consists of two main approaches: hierarchical decomposition analysis and multiresolution membership inconsistency analysis. The first approach provides a full description of the hierarchical structure of a network built upon its building blocks, while the second approach clarifies the structural inconsistency of a network at the individual node level.

Applying our multiresolution framework to trade networks, we identify the building blocks of international trade, which are the set of countries that remain consistent regardless of the resolution. Our method allows us to visualize the hierarchical structures built upon these building blocks, and the results align with the regional blocks of the global economy. Furthermore, we also find that there is a set of countries which are more inconsistent than others due to shared structural characteristics. By multiple regression analysis with various economic and political indicators, we demonstrate that there is a positive correlation between the external instability of countries and their structural inconsistency in trade networks.

## Methods

### A multiresolution framework for community structure analysis

In this paper we use the generalized Louvain algorithm ^15^, a variant of the Louvain algorithm ^16^, which detects the optimal division of a network by maximizing a generalized modularity function for a weighted network of the size *N* given by

1 $$\begin{aligned} Q = \frac{1}{2m} \sum _{i,j} \left( A_{ij}-\gamma \frac{k_i k_j}{2m} \right) \delta (g_i,g_j), \end{aligned}$$

where $A_{ij}$ is the weight of edges between nodes *i* and *j* $(i,j = 1,2,\dots ,N)$, $k_i = \sum _j A_{ij}$ is the sum of weights, or strength, of node *i*, $m = \frac{1}{2}\sum _i k_i$ is the total weight of the network, $g_i$ is an index of the community that node *i* belongs, $\gamma$ is a resolution parameter that can be adjusted to detect community structures in multiple scales, and $\delta$ is the Kronecker delta. In our multiresolution framework, we define the resulting division from a realization of stochastic community detection algorithm as a configuration, where the number of communities in the configuration for a given resolution $\gamma$ is $n_\gamma$ and $g_i \in \{1,\dots ,n_\gamma \}$. The collection of configurations in each resolution $\gamma$ is defined as an ensemble $C_\gamma$.

In theory, the possible choice of $\gamma$ ranges from zero to infinity. Therefore, it is necessary to decide a meaningful range of $\gamma$ for practical application. We first measure the average number of communities for each resolution $\gamma$ such that

2 $$\begin{aligned} \langle n_\gamma \rangle = \frac{1}{\vert C_\gamma \vert } \sum _{\alpha \in C_\gamma } n_\gamma ^\alpha , \end{aligned}$$

where $\alpha$ is an index for a configuration and $\vert C_\gamma \vert$ is the number of configurations in the ensemble for resolution $\gamma$ and is equivalent to the number of realizations for which the community detection algorithm runs at a given resolution. The minimum resolution $\gamma _{\rm min}$ can be given by the minimum level of $\gamma$ that satisfies $\langle n_\gamma \rangle > 1$ because the range of resolutions where all nodes belong to a single community is irrelevant for the analysis of community structure.

On the other hand, at a high value of $\gamma$, the number of communities becomes nearly as many as the number of nodes. Clearly, configurations with the fragments of individual nodes are difficult to be considered as a meaningful representation of community structure. The appropriate limit of the smallest community scale would vary depending on the structural characteristic of a given network, but testing on various model networks, we find that setting $\gamma _{\rm max}$ to be the maximum level of $\gamma$ that satisfies $\langle n_\gamma \rangle \le N/3$ serves the goal of our framework.

### Hierarchical decomposition analysis

The hierarchical decomposition analysis aims to aggregate configurations from different resolutions and elucidate the underlying structure of a given network. To begin with, we find the set of nodes with consistent co-memberships by measuring the cooccurrence $\phi _{ij}(\gamma )$ or the proportion of configurations in the ensemble for resolution $\gamma$ where nodes *i*, *j* are assigned to the same community:

3 $$\begin{aligned} \phi _{ij}(\gamma ) = \langle \delta (g_i^\alpha ,g_j^\alpha ) \rangle \equiv \frac{1}{\vert C_\gamma \vert } \sum _{\alpha \in C_\gamma } \delta (g_i^\alpha ,g_j^\alpha ), \end{aligned}$$

where $g_i^\alpha$ is the community membership index of node *i* for configuration $\alpha$. In Eq. (3), $\delta (g_i^\alpha ,g_j^\alpha )=1$ if nodes *i*, *j* share the same community membership in configuration $\alpha$, and $\delta (g_i^\alpha ,g_j^\alpha )=0$ otherwise. Accordingly, the cooccurrence $\phi _{ij}(\gamma )$ ranges from zero to unity. In order to extend this approach for different resolutions, we define the set of all ensembles from the range of resolutions as a multiresolution ensemble. Then, the multiresolution cooccurrence $\Phi _{ij}$, is given by

4 $$\begin{aligned} \Phi _{ij}= \langle \phi _{ij}(\gamma ) \rangle _\gamma , \end{aligned}$$

where $\langle \dots \rangle _\gamma$ is an average over all values of $\gamma$. This quantity measures how consistently nodes *i*, *j* maintain their comembership throughout resolutions.

**Figure 1:**
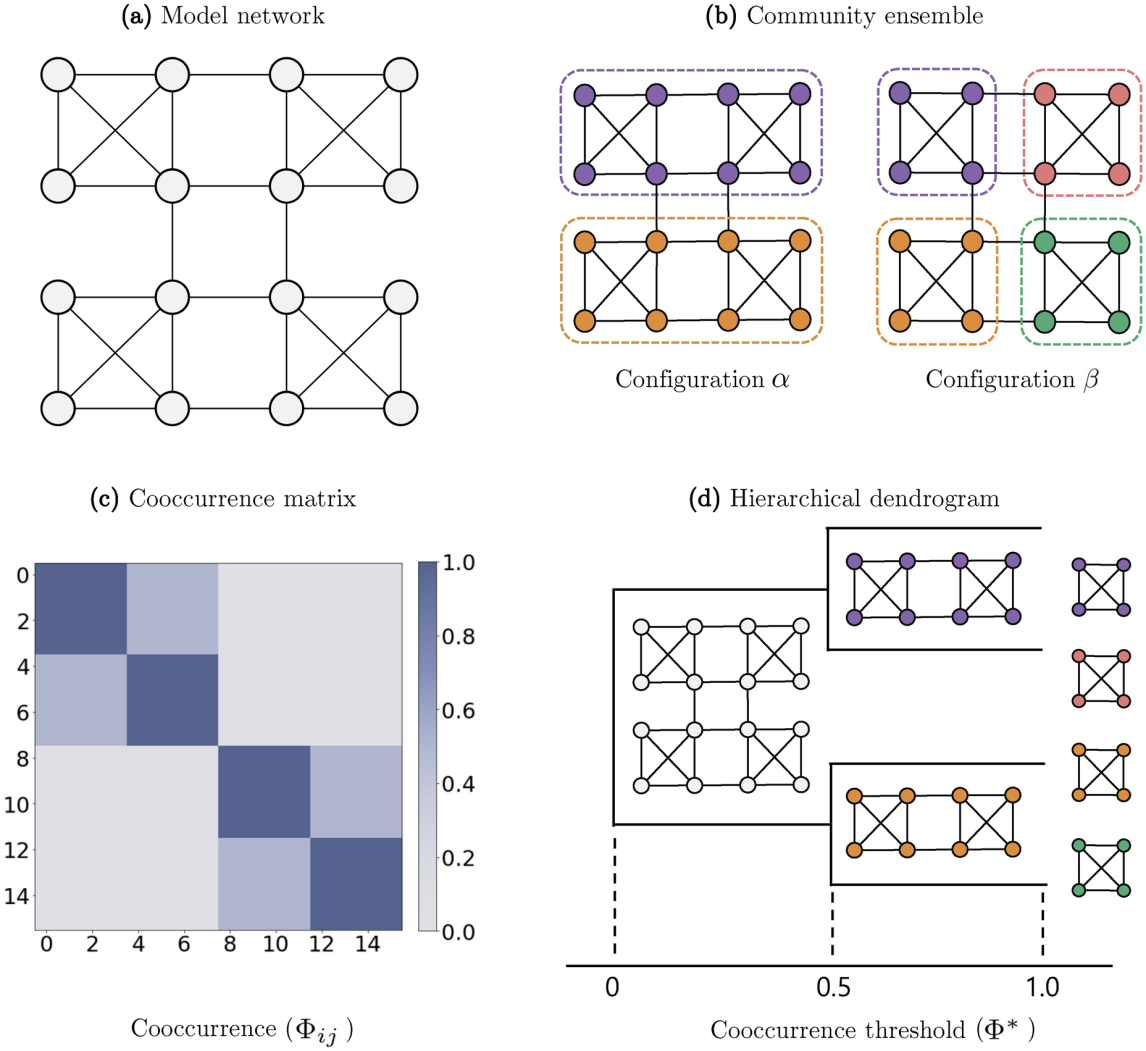
An illustrative example of the hierarchical decomposition analysis. (**a**) A representation of the model network of size 16. (**b**) A representation of the two configurations in the community ensemble of the model network. (**c**) A cooccurrence matrix constructed from the ensemble. The blocks are colored dark blue, light blue, and gray for ${{\Phi}_{ij}} = 1$, 0.5 and 0 respectively. (**d**) A dendrogram built by the hierarchical decomposition. The horizontal axis refers to the cooccurrence threshold $\Phi ^*$. For each merging point of branches, a configuration of the corresponding component is illustrated.

For instance, suppose that a model network of size 16 in Fig. 1a is given and a multiresolution ensemble with two configurations shown in Fig. 1b is obtained from the network; in configuration $\alpha$, two communities of eight nodes are detected, which are respectively subdivided into two communities of four nodes in configuration $\beta$. The multiresolution cooccurrence matrix is shown in Fig. 1c, of which the elements are $\Phi _{ij}$. The values of $\Phi _{ij}$ are represented as shades of color. The dark blue-colored block diagonals represent the four communities in configuration $\beta$ in Fig. 1b, and their the members in each community consistently share the same community membership in the ensemble ($\Phi _{ij}=1$). The light blue-colored areas stand for the pairs of nodes that share the comembership only in configuration $\alpha$ in Fig. 1b ($\Phi _{ij}=0.5$). The nodes that were never been assigned together are colored in light gray ($\Phi _{ij}=0$).

The following step is to use the multiresolution cooccurrence matrix as an adjacency matrix to reconstruct the network into a complete weighted network where the weight of each edge corresponds to the cooccurrence $\Phi _{ij}$. Then, given a threshold $\Phi ^* \in [0,1]$, we remove all the edges with the weight $\Phi _{ij} < \Phi ^*$ from the reconstructed network and find a configuration of connected components. With $\Phi ^*$ increasing from zero to unity, the fully-connected network becomes gradually decomposed into smaller components.

As shown in Fig. 1d, we can illustrate the entire composition of a network with a dendrogram. The horizontal axis refers to the cooccurrence threshold $\Phi ^*$, and the configuration of each corresponding component is presented for every merging point of branches. In this case, the components or blocks of nodes described on the dendrogram accord with the representative communities detected in a certain level of resolution.

However, it is not necessarily the case in networks with more complicated structure. The components of the dendrogram, particularly those with the highest level of $\Phi ^*$, may not correspond to communities themselves. Indeed, they are the building blocks of community structure which can be put together to assemble the detected communities. These blocks of nodes maintain their comemberships with high consistency throughout the change of resolutions and can be assembled to constitute all the communities from an ensemble. The dendrogram provides a description of the hierarchical structure the building blocks construct.

### Multiresolution membership inconsistency analysis

In the preceding section a method for decomposing a network into blocks of nodes based on their consistencies in a multiresolution ensemble is described. In this section we extend our multiresolution framework to analyze the structure of a network in the individual node level by measuring the consistency of communities for each node.

In order to quantify the node-level consistency, we employ a measure called membership inconsistency (MeI) proposed by Lee, *et al.* ^14^. First, let us denote a set of nodes which share community membership with node *i* in each configuration $\alpha$ by $\psi _i ^ \alpha$ such that

5 $$\begin{aligned} \psi _i^\alpha = \{ j \mid g_j^\alpha = g_i^\alpha \} \end{aligned}$$

where $g_i^\alpha$ refers to the community to which node *i* is assigned in configuration $\alpha$. The consistency of node *i* depends on how similar $\psi _i^\alpha$ is between all the configurations in a multiresolution ensemble. The similarity between sets $\psi _i^\alpha$ and $\psi _i^\beta$ is given by

6 $$\begin{aligned} J_i^{\alpha \beta }= \frac{\vert \psi _i^\alpha \cap \psi _i^\beta \vert }{\vert \psi _i^\alpha \cup \psi _i^\beta \vert } \end{aligned}$$

which is the Jaccard similarity coefficient. The maximum value of $J_i^{ \alpha \beta }$ occurs when $\psi _i^{\alpha }$ is equivalent to $\psi _i^ \beta$, where $J_i^{\alpha \beta }=1$. However, note that $J_i^{\alpha \beta }$ cannot be zero, for $\psi _i^\alpha$ and $\psi _i^\beta$ always share at least one node, node *i* itself. Using the Jaccard coefficient, the MeI of node *i* in a multiresolution ensemble, or multiresolution membership inconsistency, is defined as

7 $$\begin{aligned} { \Psi _i = \Biggl ( \biggl \langle \frac{1}{\vert C_\gamma \vert ^2} \sum _{\alpha ,\beta \in C_\gamma }J_i^{\alpha \beta } \biggr \rangle _\gamma \Biggl )^{-1} } \end{aligned}$$

where $C_\gamma$ is a configuration ensemble for resolution $\gamma$. The minimum value of $\Psi _i$ is unity, where the community of node *i* is perfectly consistent for all the configurations in an ensemble.

**Figure 2:**
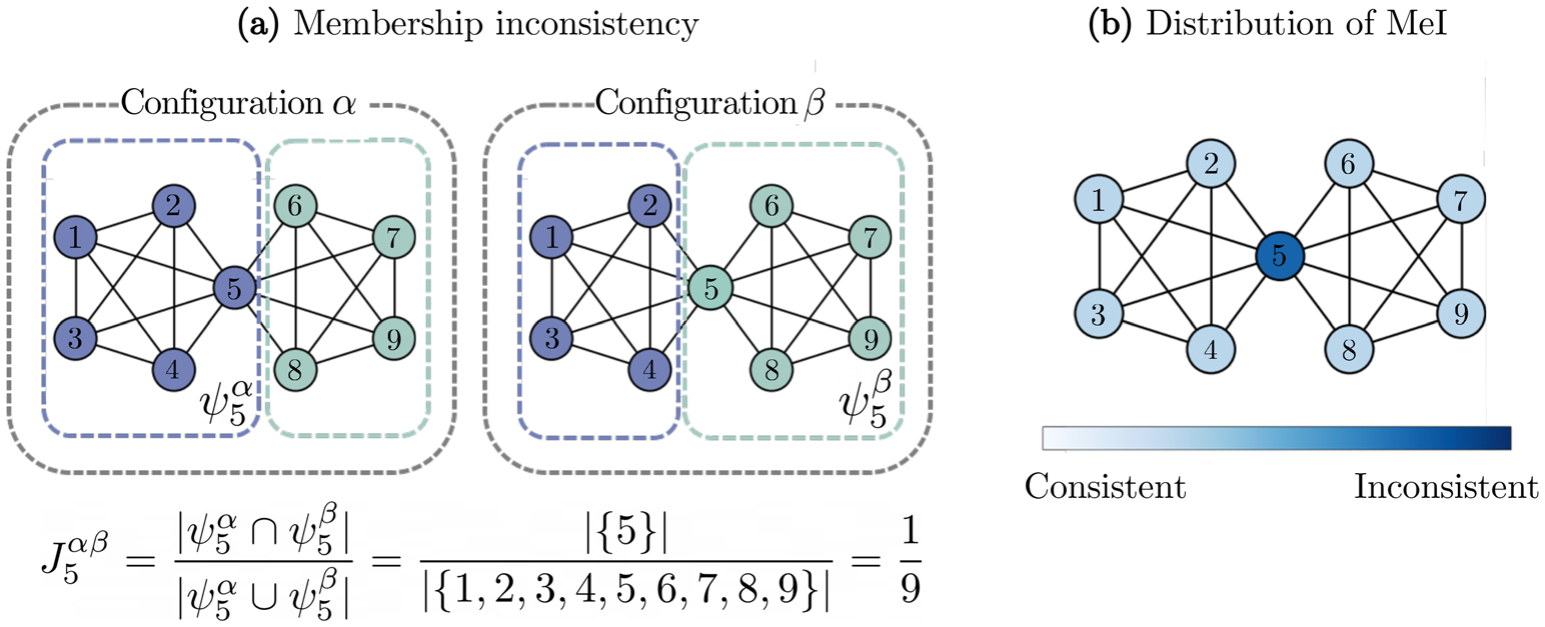
An illustrative example of the membership inconsistency analysis. (**a**) A representation of the two configurations $\alpha$ and $\beta$ of the example network. From each configuration, the set of nodes that belong to the same community with node 5 is given by $\psi _{5}^{\alpha }$ and $\psi _{5}^{\beta }$ respectively. (**b**) An illustration of the distribution of $\Psi$ of the network. The colors of the nodes indicate the relative strengths of MeI. A node with higher $\Psi$ is colored in darker blue.

An illustrative example of measuring the MeI value is given in Fig. 2a. In this network there are two cliques of five nodes, and the node in the middle (node 5) constitutes the overlap of the cliques. Applying community detection algorithms to this network yields two different configurations where node 5 alternates between two completely distinct communities ($\vert \psi _5^\alpha \cap \psi _5^\beta \vert =1$). The distribution of MeI is illustrated in Fig. 2b. The colors of the nodes indicate the relative strengths of MeI, and we can clearly notice that node 5 in the middle shows the most inconsistent community membership.

## Results

### Hierarchical decomposition of trade networks

In this study we use the historical trade data from the Trade Map database of International Trade Centre for the period 2010–2019 ^17^. We build an undirected weighted network of 45 countries based on the data, using annual bilateral trade volume as a weight between each pair of countries. A detailed description of bilateral trade volume and additional experiments with the expanded scope of countries are provided in the supplementary material.

We apply the generalized Louvain algorithm ^15^ for community detection to the international trade networks. For the resolution parameter $\gamma \in \{\gamma _{\textrm{min}}, \gamma _{\textrm{min}}+\Delta \gamma , \cdots , \gamma _{\textrm{max}}\}$, $\gamma _{\textrm{min}}$ is fixed at the minimum level of $\gamma$ that satisfies $\langle n_\gamma \rangle > 1$, $\gamma _{\textrm{max}}$ is set to be the maximum level of $\gamma$ that satisfies $\langle n_\gamma \rangle \le N/3$, and $\Delta \gamma =0.01$. For each resolution, a community ensemble is formed by 1000 independent community detection results. By aggregating the configurations from the range of resolutions, we construct a multiresolution ensemble and conduct a hierarchical decomposition analysis by measuring the multiresolution cooccurrence $\Phi _{ij}$ in Eq. (4).

**Figure 3:**
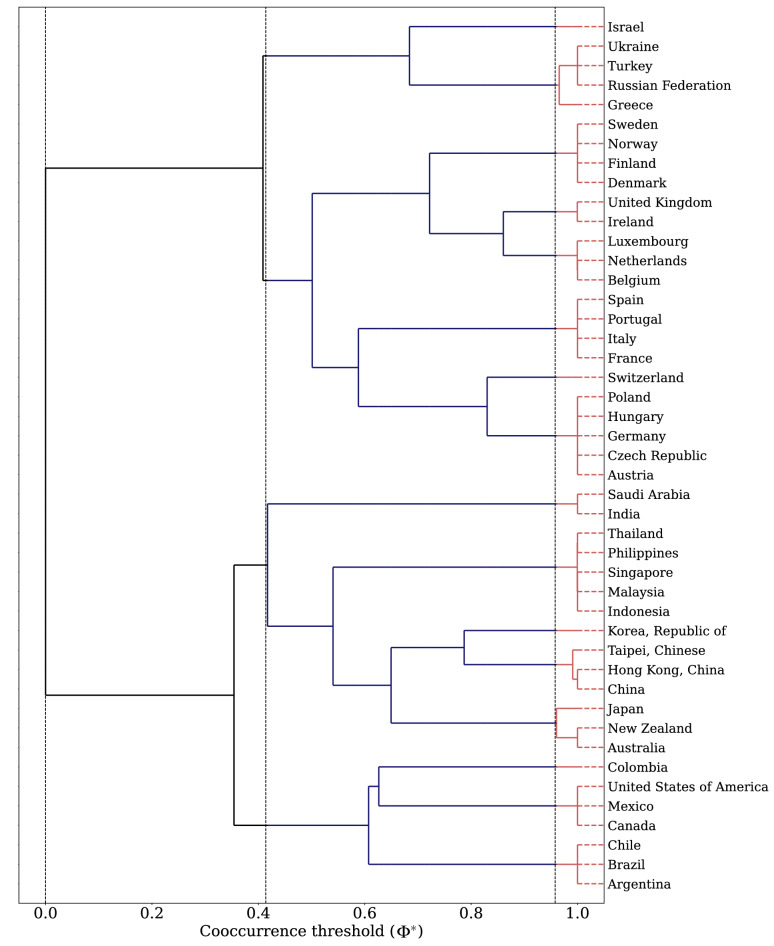
The dendrogram of the international trade network in 2018 constructed by the hierarchical decomposition analysis. The horizontal axis refers to the cooccurrence threshold $\Phi ^*$. The vertical dashed lines are drawn for $\Phi ^*=0$, 0.41 and 0.96, which correspond to Fig. 4a–c respectively.

**Figure 4:**
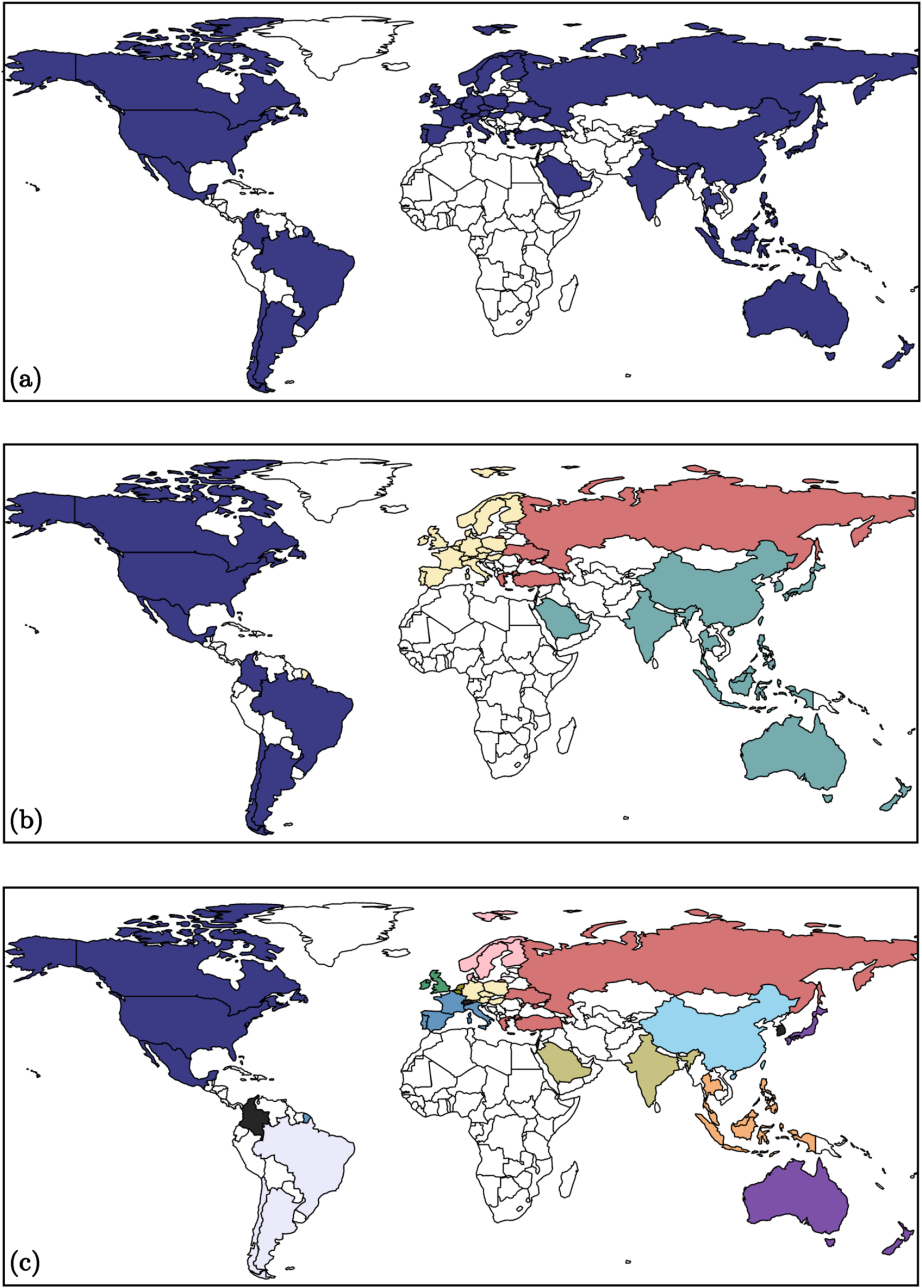
The configurations of countries at the selected cooccurrence thresholds illustrated by the world map. (**a**) The configuration at $\Phi ^*=0$. (**b**) The configuration at $\Phi ^*=0.41$. (**c**) The configuration at $\Phi ^*=0.96$. Different colors represent different components except the following cases; the countries that do not belong to a component with any other countries are colored in black, and the unselected countries are colored in white.

The results of the hierarchical decomposition analysis are presented in Figs. 3 and 4. Figure 3 is a dendrogram that describes how the international trade network splits into smaller components with an increase of the cooccurrence threshold $\Phi ^*$. Figure 4a–c illustrate the maps of divisions at $\Phi ^*=0$, 0.41, 0.96, respectively. Starting from a single large component at $\Phi ^*=0$ in Fig. 4a, the countries are divided into four components based on the continent as $\Phi ^*$ is increased: Western Europe, Eastern Europe, Asia and America (Fig. 4b). After multiple steps of splits, the countries are partitioned into 16 groups (Fig. 4c). Each group consists of the countries that consistently sustained comembership over most of the resolutions, which represent the building blocks of international trade networks. Note that these resolution-invariant blocks shown in Fig. 4c accord with the regional blocs of the global economy. This implies that despite the deepening integration of world economies through globalization, the trading system still largely relies on the regional economic communities, which are likely influence by geography and historical patterns.

It is worth noting that the detection of communities in our framework relies on a null model, expressed as $k_i k_j/2m$ in Eq. (1), which determines the expected trade volume between each pair of countries based on their magnitudes of total trade. When the actual trade volume between two countries exceeds what is expected from the null model, the modularity between them is positive, indicating a potential presence of a community. This approach allows us to identify the underlying community structure of international trade based on relatively small magnitudes of local interactions, which may not be apparent from the raw analysis of original trade data. For instance, while the South American block composed of Chile, Brazil, and Argentina may seem like an obvious economic community, it is not very evident in the trade data itself; 42.4% of their total trade is accounted for by their trade with the US and China, whereas the total trade between these three countries accounts for only 6.4%. This demonstrates that, while the largest countries such as the US, China, and Germany take a significant share of world trade, regional economic communities still play important roles as building blocks of international trade.

Overall, visualizing the hierarchical structure of the international trade network as shown in Figs. 3 and 4 allows for a clearer understanding of the complex and multi-layered relationships and patterns within the network. It can provide valuable insights into how different regions and countries are interconnected through trade. Additionally these results highlight the importance of considering the resolution-invariant blocks identified in the analysis when studying the structure and dynamics of the international trade network.

### Multiresolution membership inconsistency of trade networks

**Figure 5:**
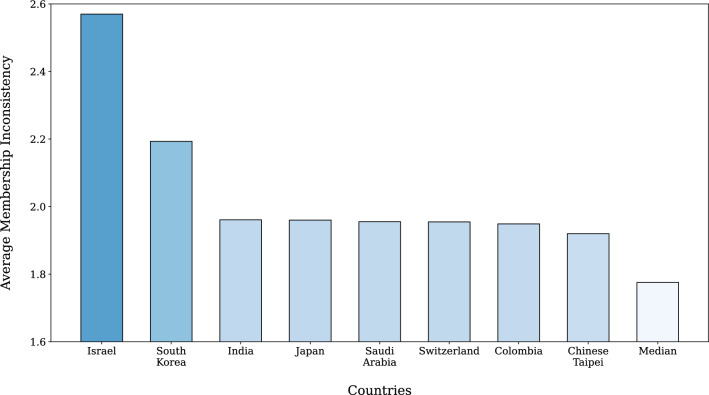
The bar chart of the average membership inconsistency (MeI) of the eight countries with the highest MeI. The MeI is averaged for the period 2010–2019. The bars of the countries with higher average MeI are colored in darker blue.

By using the multiresolution ensemble, we measure the multiresolution membership inconsistency (MeI) of each country in Eq. (7). The multiresolution MeI value $\Psi$ in trade networks is a structural attribute of each country that represents the inconsistency between the compositions of trade communities that it belongs to. For example, suppose that a country is classified to be the same group with the United States in one configuration and with China in the other. If there exist few intersections between those two groups, the country will exhibit a high level of $\Psi$. The countries with the highest $\Psi$ are presented in Fig. 5. In particular, Israel and South Korea have shown distinctly high $\Psi$ throughout a decade.

Although we can interpret the meaning of MeI in terms of network topology and find the set of countries with the higher structural inconsistency than others, it is not clear how this measure may be relevant in a real-world context. Previous research by Kim et al. ^18^ attempted to examine the relationship between the network topological characteristics of a system and its physical properties. Using community detection methods on the Chilean power grid, they found that the community consistency, a similar measure to MeI that quantifies how consistent community detection results are, determines the contribution of a node to the stability of the electricity supply of a power grid.

In the present study, we propose a hypothesis that MeI influences the instability of a country in terms of external intervention. The rationale for this hypothesis is that countries with membership in multiple economic communities may be confronted with conflicting interests between these communities ^19^. This instability can be further exacerbated by the recent trend of trade being weaponized as a tool of strategic and political influence ^20^. For instance, South Korea, which exhibits high $\Psi$, has both important economic ties with China and a critical alliance with the United States. As the rivalry between these two countries intensifies, South Korea may face political pressures from the two largest powers and struggles to determine its strategic direction for trade and foreign policy ^21^.

To test our hypothesis, we conduct a multiple regression analysis of MeI along with other political and economic factors. As a measure for instability from external intervention, we use the *External Intervention Indicator* (EII) published by the Fund for Peace ^22^. The EII estimates the influence and impact of political and economic engagement of external actors in functioning of a country, and its score ranges from 0 (the least influenced) to 10 (the most influenced). In addition, three control variables are considered in estimating the impact of MeI (independent variable) on EII dependent variable). These include the following:

- *GDP*: Economic wealth of a country is intimately connected with its power and status in contemporary politics and functions as a powerful ordering principle in international governance ^23^. A standard measure of a country’s economic wealth is Gross Domestic Product or GDP, and countries with high GDP are expected to be more resistant to external intervention. We control for this effect by including annual GDP (in trillions of current U.S. dollars) from the World Bank database ^24^.
- *Political Stability*: A country’s political stability is an important factor that influences its foreign policy behavior ^25^, and unstable political situations often provide an opportunity for external intervention ^26^. We control for this effect by including the Political Stability Index (PSI) provided by the World Bank ^27^. The PSI is a composite measure that reflects the likelihood of a disorderly transfer of government power, social unrest, as well as ethnic, religious or regional conflicts, ranging from $-2.5$ (the most unstable) to 2.5 (the most stable).
- *Trade Openness*: High dependence on trade can make countries more vulnerable to external political pressure. In particular, the asymmetrical economic dependence between two partners compromises the foreign policy behavior of the more dependent ^28^. We consider the effect of trade dependence by including trade openness (% of GDP), which is defined as the ratio of exports plus imports over GDP ^29^.

Table 1 presents the results of the regression model of the data for the period 2010–2019. The impact of MeI on EII is statistically significant ($<0.1\%$), which corresponds to our hypothesis postulating that higher membership inconsistency in the community structure of trade networks leads to higher instability of a country in terms of external intervention. GDP and PSI also yield significant estimates as previously assumed. However, note that the effect of trade openness turns out to be not statistically significant. This is an unanticipated result as the dependence on trade is considered as an important factor that influences the vulnerability of countries to external shocks, particularly in contemporary politics where trade is being weaponized for strategic influence ^20,30^. One possible explanation is that simply measuring a share of trade in a country’s economy without consideration of network topology fails to capture the complex interdependencies that affect economic and political behavior. This underlines the importance of understanding the community structure of trade networks and extracting the structural attributes of countries.

**Table 1:** The results of regression analysis. Dependent variable: External Intervention Indicator. ***$p<0.1\%$.

**Variable**		**Coefficient**	Prob. ($\mathbf {P>|t|}$)
(Intercept)		1.899***	$<0.001$
Membership Inconsistency (MeI)		1.070***	$<0.001$
Gross Domestic Product (GDP)		$-0.113$***	$<0.001$
Political Stability Index (PSI)		$-1.884$***	$<0.001$
Trade Openness		0.001	0.129
R	0.700	Prob. (F-statistic)	$<0.001$
Adjusted R$^2$	0.697	N	430

## Discussion

This study presents a multiresolution framework for analyzing international trade networks, which allows for the hierarchical decomposition of trade communities and facilitates a more in-depth understanding of the relationships between countries through effective visualization. Our community-based approach effectively demonstrates that the building blocks of international trade align with the regional blocks of the world economy, which is not apparent from the raw analysis of original trade data due to the relatively small magnitude of local trade compared to that of the largest economies.

In addition, we introduce a metric called multiresolution membership inconsistency and investigate its correlation with a country’s vulnerability to external intervention. Our results suggest that countries with structural inconsistency in their trade network are more susceptible to external influence. This may be because conflicting interests between multiple economic communities can destabilize diplomatic relations and make the country more vulnerable to external intervention, particularly in the current international political climate, where trade is increasingly being used as a weapon for strategic and political influence.

It is important to note that this study has a limitation in that it is based on a snapshot of the trade network at a specific point in time and does not reflect the dynamics and evolution of trade communities over time. To address this limitation, future research could consider conducting longitudinal analyses of the trade network, tracking changes in community structure and their relationship with economic and political indicators.

Overall, our findings demonstrate the importance of considering different resolutions when analyzing complex interdependencies within trade networks and suggest that structural attributes in networks may serve as an effective measure for complementing existing social science measures. The development of new metrics for international trade based on network science presents new opportunities for accurately evaluating the characteristics and behaviors of countries in both economic and political contexts. Further research is needed to fully explore the potential applications of our findings.

## Supplementary Information

Supplementary Information.

## Data Availability

The real datasets analyzed during the present study are available at https://www.trademap.org/.
