# Supplementary material for: A multiresolution framework for the analysis of community structure in international trade networks
Source: Sci Rep. 2023 Apr 7;13:5721. doi: 10.1038/s41598-023-32686-2 (PMC10082076; doi:10.1038/s41598-023-32686-2)
Supplement: Supplementary file 1 — Supplementary Information. [file 41598_2023_32686_MOESM1_ESM.pdf]

Supplementary material: A multiresolution framework for the analysis of community structure in international trade networks

Wonguk Cho[1,2], Daekyung Lee[3], and Beom Jun Kim[2,*]

[1]Graduate School of Data Science, Seoul National University, Seoul 08826, Republic of Korea
[2]Department of Physics, Sungkyunkwan University, Suwon 16419, Republic of Korea
[3]Department of Energy Technology, Korea Institute of Energy Technology, Naju 58322, Republic of Korea
[*]beomjun@skku.edu

A detailed description of bilateral trade volume

Bilateral trade volume refers to the total value of all products exchanged between two countries, measured in one thousand US dollars. While the summation of a country's imports from all other countries should theoretically be equal to the summation of exports to that country, there can be some discrepancies in the data due to differences in measurement methods and timing. To ensure consistency in our analysis, we use import data to calculate the bilateral trade volume. Specifically, we calculate the bilateral trade volume between country A and country B as the sum of country A's imports from country B and country B's imports from country A. This approach helps to account for any differences that may exist between import and export data.

Additional experiments

To provide a more comprehensive analysis with a wider range of countries, we conduct additional studies using trade data from total 65 countries with the highest average trade volume for the period from 2010 to 2019. These top 65 countries account for around 99% of the world's trade volume. We build an undirected weighted network of these countries using annual bilateral trade volume as a weight between each pair of countries and conduct the hierarchical decomposition analysis and the multiresolution membership inconsistency analysis. Figure 1 shows the dendrogram of the international trade network constructed by the hierarchical decomposition analysis. Figure 2 displays the configurations of countries at the selected cooccurrence thresholds illustrated by the world map. As Φ^* is increased, we observe that the countries are gradually divided into regional economic blocs. Figure 3 presents the bar chart of the average membership inconsistency (MeI) of the countries with the highest MeI. Table 1 is the result of the multiple regression analysis of MeI along with other political and economic factors. These results show that the specific rankings of MeI for some countries may change by the inclusion of additional countries, but the overall statistical significance and direction of correlation remain consistent. Overall, our main findings remain consistent despite the expanded scope of countries considered.

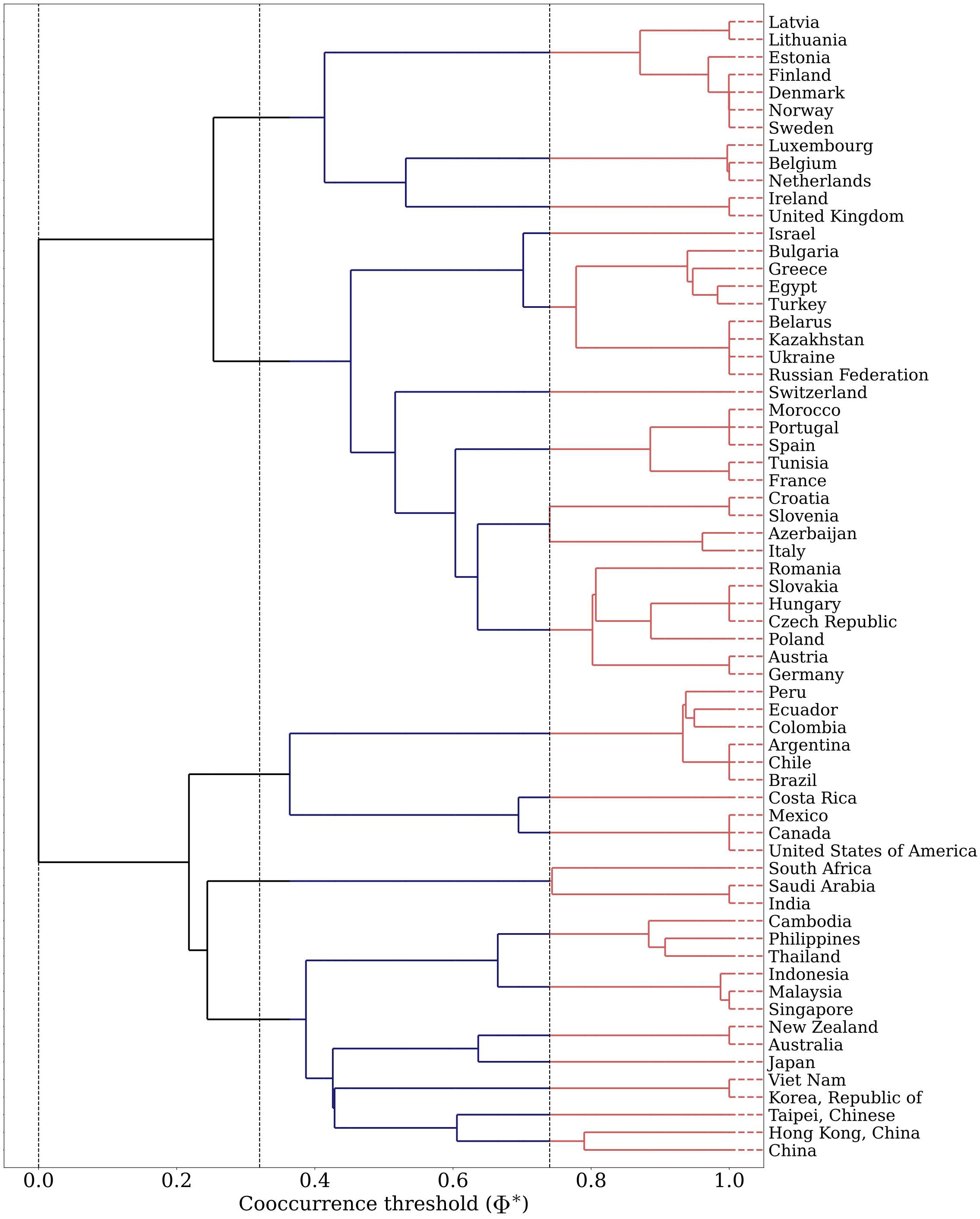

Figure 1. The dendrogram of the international trade network in 2018 constructed by the hierarchical decomposition analysis. The horizontal axis refers to the cooccurrence threshold Φ^*. The vertical dashed lines are drawn for $\Phi^* = 0$, 0.32 and 0.74, which correspond to Fig. 2a, 2b, and 2c respectively.

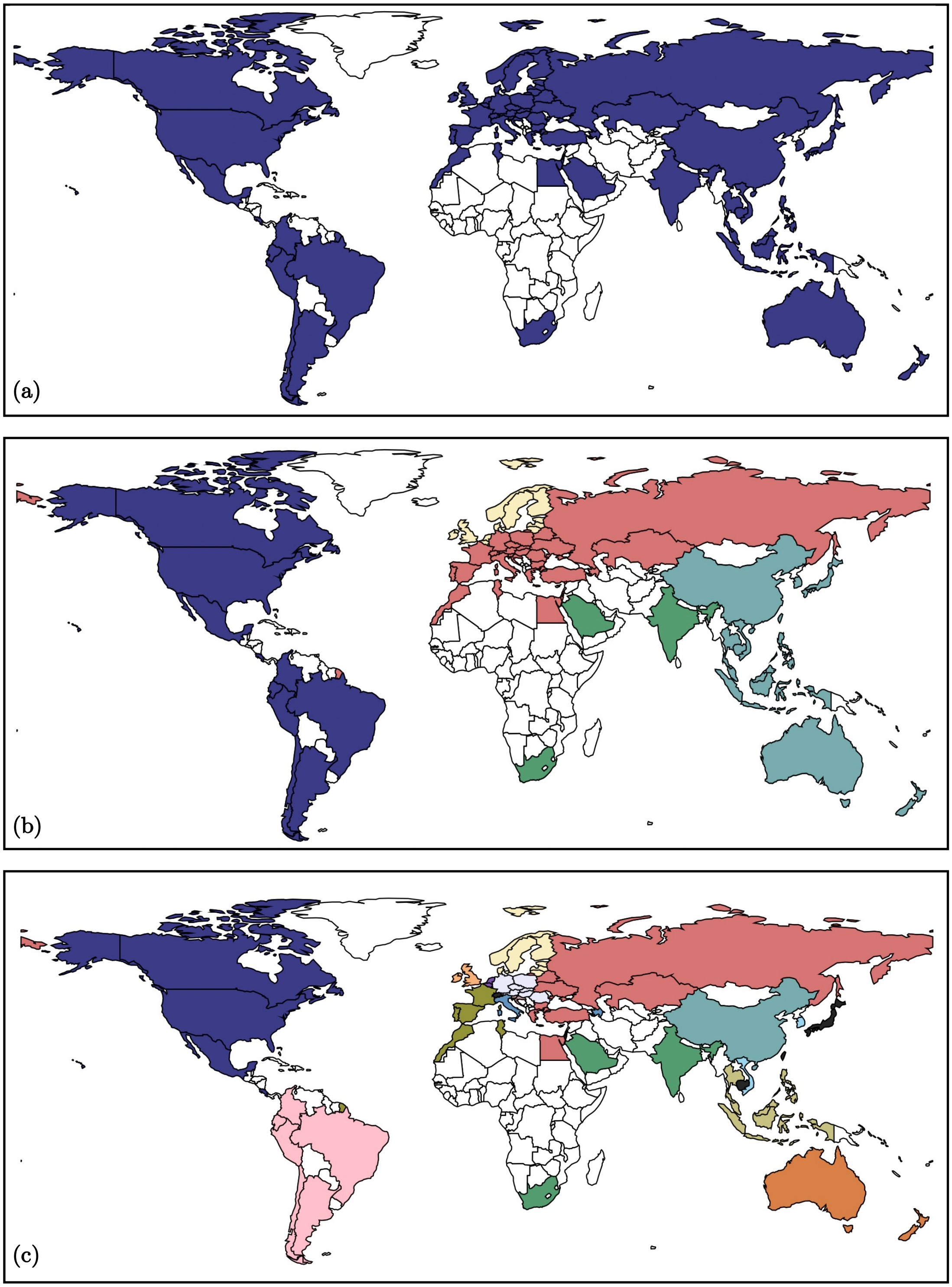

Figure 2. The configurations of countries at the selected cooccurrence thresholds illustrated by the world map. (**a**) The configuration at $\Phi^* = 0$. (**b**) The configuration at $\Phi^* = 0.32$. (**c**) The configuration at $\Phi^* = 0.74$. Different colors represent different components except the following cases; the countries that do not belong to a component with any other countries are colored in black, and the unselected countries are colored in white.

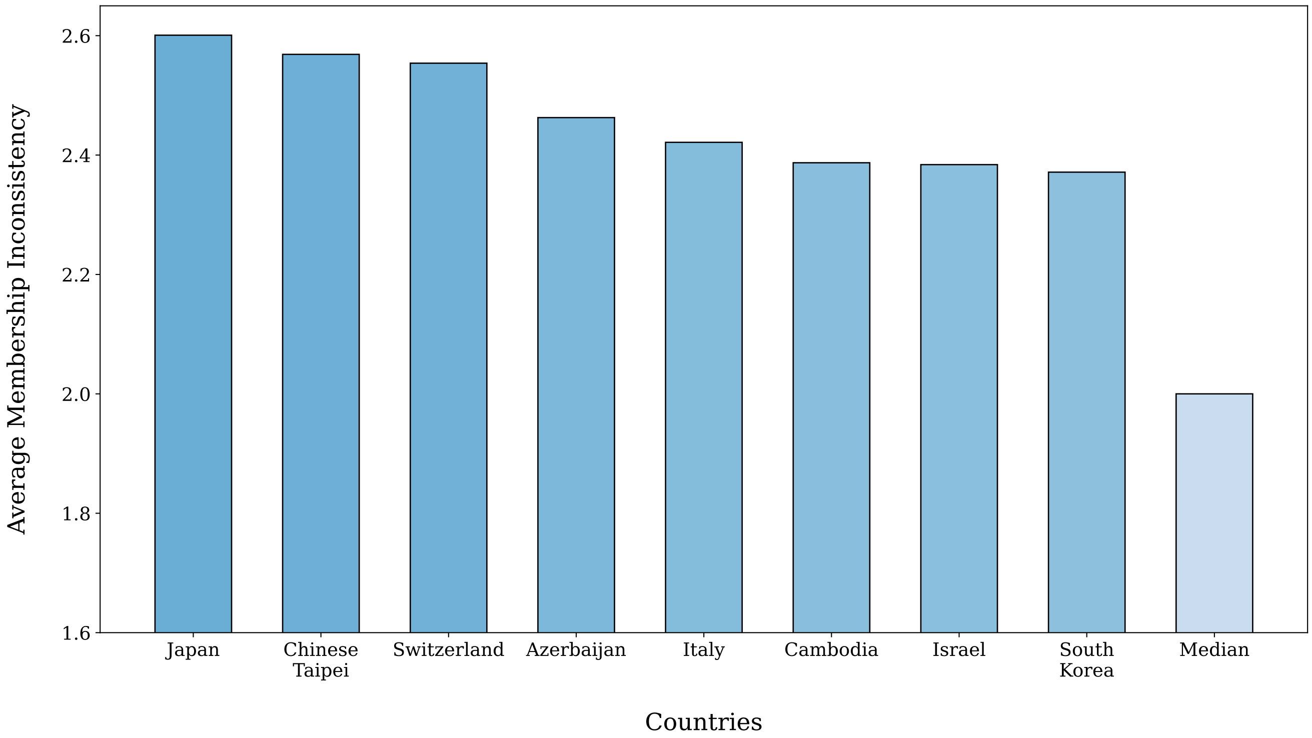

Figure 3. The bar chart of the average membership inconsistency (MeI) of the eight countries with the highest MeI. The MeI is averaged for the period 2010-2019. The bars of the countries with higher average MeI are colored in darker blue.

Table 1. The results of regression analysis. Dependent variable: External Intervention Indicator. $^{**}p < 1\%$, $^{***}p < 0.1\%$.

Variable		Coefficient	Prob. (P > |t|)
(Intercept)		3.272***	< 0.001
Membership Inconsistency (MeI)		0.458**	0.008
Gross Domestic Product (GDP)		−0.166***	< 0.001
Political Stability Index (PSI)		−1.984***	< 0.001
Trade Openness		0.002	0.019
R	0.632	Prob. (F-statistic)	< 0.001
Adjusted R^2	0.630	N	620
